# Discovery of TRPV4-Targeting Small Molecules with Anti-Influenza Effects Through Machine Learning and Experimental Validation

**DOI:** 10.3390/ijms26031381

**Published:** 2025-02-06

**Authors:** Yan Sun, Jiajing Wu, Beilei Shen, Hengzheng Yang, Huizi Cui, Weiwei Han, Rongbo Luo, Shijun Zhang, He Li, Bingshuo Qian, Lingjun Fan, Junkui Zhang, Tiecheng Wang, Xianzhu Xia, Fang Yan, Yuwei Gao

**Affiliations:** 1 College of Veterinary Medicine, Shanxi Agricultural University, Jinzhong 030801, China; 15353739367@163.com; 2 State Key Laboratory of Pathogen and Biosecurity, Key Laboratory of Jilin Province for Zoonosis Prevention and Control, Changchun Veterinary Research Institute, Chinese Academy of Agricultural Sciences, Changchun 130122, China; wujiajing1996@163.com (J.W.); shencn2023@sina.com (B.S.); luorb0305@163.com (R.L.); zsj202328@163.com (S.Z.); lihetcm01@163.com (H.L.); bensonqian0@gmail.com (B.Q.); fanlingjun0516@163.com (L.F.); 104753221395@henu.edu.cn (J.Z.); wgcha@163.com (T.W.); xiaxzh@cae.cn (X.X.); 3 Key Laboratory for Molecular Enzymology and Engineering of Ministry of Education, School of Life Science, Jilin University, Changchun 130012, China; yanghz24@mails.jlu.edu.cn (H.Y.); hzcui23@mails.jlu.edu.cn (H.C.); weiweihan@jlu.edu.cn (W.H.); 4 Jiangsu Co-Innovation Center for Prevention and Control of Important Animal Infectious Diseases and Zoonoses, Yangzhou University, Yangzhou 225009, China

**Keywords:** TRPV4, anti-influenza, H1N1, machine learning, repurposing drugs, molecular docking

## Abstract

Transient receptor potential vanilloid 4 (TRPV4) is a calcium-permeable cation channel critical for maintaining intracellular Ca^2+^ homeostasis and is essential in regulating immune responses, metabolic processes, and signal transduction. Recent studies have shown that TRPV4 activation enhances influenza A virus infection, promoting viral replication and transmission. However, there has been limited exploration of antiviral drugs targeting the TRPV4 channel. In this study, we developed the first machine learning model specifically designed to predict TRPV4 inhibitory small molecules, providing a novel approach for rapidly identifying repurposed drugs with potential antiviral effects. Our approach integrated machine learning, virtual screening, data analysis, and experimental validation to efficiently screen and evaluate candidate molecules. For high-throughput virtual screening, we employed computational methods to screen open-source molecular databases targeting the TRPV4 receptor protein. The virtual screening results were ranked based on predicted scores from our optimized model and binding energy, allowing us to prioritize potential inhibitors. Fifteen small-molecule drugs were selected for further in vitro and in vivo antiviral testing against influenza. Notably, glecaprevir and everolimus demonstrated significant inhibitory effects on the influenza virus, markedly improving survival rates in influenza-infected mice (protection rates of 80% and 100%, respectively). We also validated the mechanisms by which these drugs interact with the TRPV4 channel. In summary, our study presents the first predictive model for identifying TRPV4 inhibitors, underscoring TRPV4 inhibition as a promising strategy for antiviral drug development against influenza. This pioneering approach lays the groundwork for future clinical research targeting the TRPV4 channel in antiviral therapies.

## 1. Introduction

Influenza virus (IV), belonging to the family Myxoviridae, a single negative-strand segmented RNA virus which has a wide host range, can break through the interspecific barrier and infect a variety of birds and mammals. Influenza A virus (IAV) is the most pathogenic of the four subtypes of influenza viruses, and infants and people with low immunity are susceptible to infection [1,2]. Therefore, methods of preventing influenza virus have become an important global public health issue all over the world. Antiviral drugs are one of the main treatments for influenza virus. Currently, a variety of antiviral drugs have been approved for the treatment of influenza by inhibiting neuraminidase (EC: 3.2.1.18) on the surface of the virus, such as neuraminidase inhibitor oseltamivir (Tamiflu) and zanamivir (Relenza) [3]. Neuraminidase cleaves sialic acid residues on new virus particles, aiding virus release from the cell membrane. When inhibited, sialic acid residues are not cleaved, impeding new virus particle release from infected cells and suppressing virus spread in the body [4]. However, due to the rapid mutation of the influenza virus, the effectiveness of some antiviral drugs may be limited. For example, clinical trial data showed that the I38T/F/M mutation in the polymerase acidic (PA) subunit reduced the efficacy of baloxavir and led to viral load rebound in treated patients [5]. As the virus continues to evolve, traditional pharmaceutic treatment regimens are facing unprecedented challenges. The emergence of drug resistance not only reduces the effectiveness of currently available therapeutic medications but also makes many originally effective drugs no longer reliable [6]. Accordingly, it will save a great deal of time and money for clinical research if a therapeutic candidate that can effectively suppress the virus can be mined from the approved completed medicine.

The Ca^2+^ signaling pathway mediated by TPRV4 plays a crucial role in a variety of viral infections [7]. During herpes simplex virus type 2 (HSV-2) infection, viral glycoproteins can bind to TRPV4 channels and induce a rapid and transient rise in intracellular Ca^2+^ concentration, promoting viral infection [8]. Additionally, when cells were exposed to Zika virus or purified viral envelope proteins, TRPV4 mediated Ca^2+^ influx and drove nuclear translocation of DEAD-box RNA helicase (DDX3X) (EC:3.6.4.13) [9,10]. In the process of respiratory virus infection, this channel can aggravate acute lung injury caused by virus infection by prompting innate immune cells (such as macrophages and neutrophils) to release proteases, cytokines and reactive oxygen species [11]. Targeting TRPV4 inhibitors can diminish the infectivity of herpes simplex virus, Zika virus, hepatitis C, and dengue fever [12,13]. Together, the findings indicate that TRPV4 plays an important role in regulating helicase-dependent RNA metabolism, suggesting that TRPV4 can be used as a potential antiviral host factor. Further exploration will contribute to the development of broad-spectrum antiviral drugs.

Computer aided drug design (CADD) is a method to study the interaction between drugs and receptors by means of computational chemistry, computational biology, molecular graphics, mathematical statistics, database and other technologies, with a view to discovering, designing and optimizing the methodology set of innovative drug molecules [14]. Presently, CADD with molecular simulation as its core technology has been widely used in drug design [15,16,17,18]. In recent years, machine learning techniques have emerged as powerful tools in drug discovery, aiding in the analysis of large-scale data, the prediction of biological activities, and the optimization of molecular structures. By integrating machine learning models into the CADD pipeline, the efficiency and accuracy of drug discovery processes have been significantly improved. In this study, high-throughput virtual screening, molecular docking, and machine learning predictions are applied to help discover drugs with potential antiviral capabilities.

On the basis of drug screening with TRPV4 as the target using the open-source database, the screening results were incorporated with the analysis of drug availability and antiviral effects. Herein, 15 small-molecule drugs for biological experiments were selected in vitro and in vivo. Then, two drugs were found to be candidates for anti-influenza virus. By combining machine learning models with computer simulations and biological experiments, glecaprevir and everolimus have the potential to be developed as candidates for anti-influenza virus therapy.

## 2. Result

### 2.1. Machine Learning Integration

**Figure 1:**
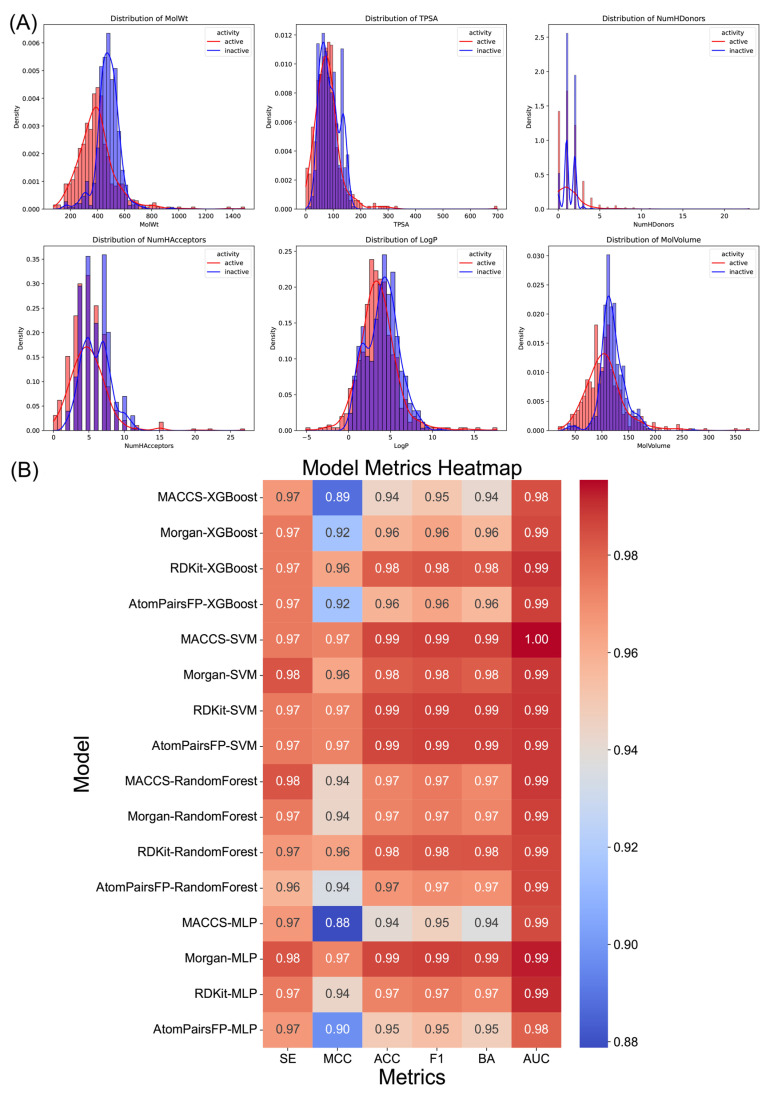
(**A**) Distributions of various molecular descriptors (MolWt, TPSA, NumHDonors, NumHAcceptors, LogP, and MolVolume) for active and inactive molecules. (**B**) The heatmap presents the performance metrics of various machine learning models using different molecular fingerprints (MACCS keys, Morgan, RDKit, and atom pairs) and classifiers (XGBoost 2.0.0, SVM, random forest, and MLP).

In this study, the distributions of molecular descriptors—molecular weight (MolWt), topological polar surface area (TPSA), number of hydrogen donors (NumHDonors), number of hydrogen acceptors (NumHAcceptors), LogP (partition coefficient), and molecular volume (MolVolume)—reveal differences between active and inactive TRPV4 inhibitors. Molecular descriptors are numerical values representing specific chemical or physical properties of a molecule. For example, TPSA quantifies the polar surface area of a molecule, calculated as the sum of the surfaces of polar atoms (typically oxygen and nitrogen, including attached hydrogens) based on the molecule’s 2D topology. LogP, representing the logarithm of a compound’s partition coefficient between n-octanol and water, indicates its hydrophobicity and balance between hydrophilic and lipophilic properties. Active compounds generally display lower molecular weights, smaller TPSA values, and fewer hydrogen donors compared to inactive compounds. These distribution patterns suggest that certain molecular properties are correlated with the activity of TRPV4 inhibitors, highlighting key molecular features to consider for identifying active molecules (Figure 1A).

In addition to molecular descriptors, molecular fingerprints were employed for machine learning model development. Unlike molecular descriptors, which quantify physicochemical properties, molecular fingerprints are binary or integer representations that encode the presence or absence of specific substructures or patterns in a molecule. Various fingerprints, including MACCS keys, Morgan, RDKit, and atom pairs, were combined with classifiers such as support vector machine (SVM), random forest (RF), multi-layer perceptron (MLP), and extreme gradient boosting (XGBoost version 2.0.0) to predict the activity of TRPV4 inhibitors. Among these models, the MACCS-SVM model demonstrated excellent performance, achieving an accuracy of 99%, with high scores in sensitivity (SE), Matthews correlation coefficient (MCC), F1 score, balanced accuracy (BA), and area under the curve (AUC), as shown in the heatmap (Figure 1B). The robust accuracy and balance of this model across various metrics indicate its reliability in distinguishing active TRPV4 inhibitors from inactive molecules, reducing the likelihood of false positives during the screening process.

**Figure 2:**
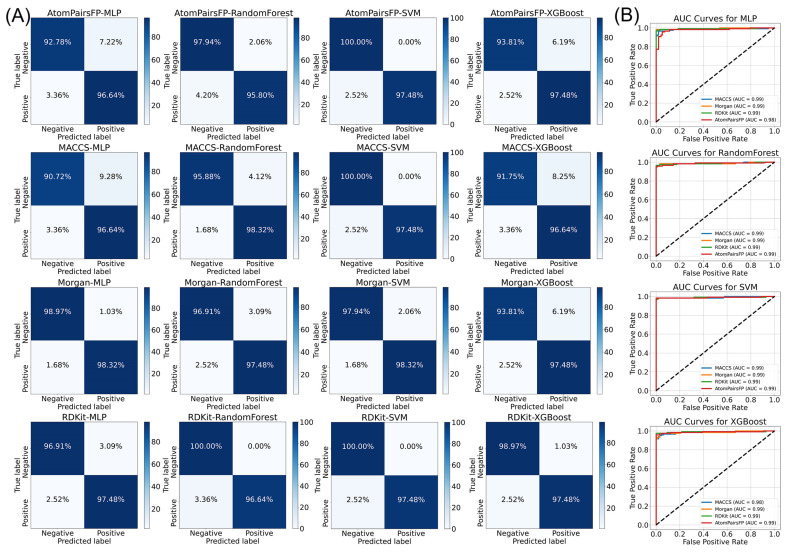
Performance evaluation of machine learning models for predicting TRPV4 inhibitor activity. (**A**) Confusion matrices of selected models show the classification performance in terms of true positives, true negatives, false positives, and false negatives. (**B**) AUC curves for each classifier (MLP, RandomForest, SVM, XGBoost 2.0.0) across different fingerprints (MACCS, Morgan, RDKit (Greg Landrum, Switzerland), AtomPairsFP), illustrating the classifiers’ ability to differentiate between active and inactive compounds.The black dashed line is random classifier.

The confusion matrices also provide insight into the predictive capability of each model. For MACCS-SVM, all negative samples were correctly classified (100% specificity), while 97.48% of positive samples were accurately identified, reflecting its ability to maintain high sensitivity and specificity. These results, along with the high MCC and AUC values, validate the MACCS-SVM model as the optimal choice for screening candidate molecules (Figure 2).

Using the MACCS-SVM model, we conducted high-throughput virtual screening on 170,000 molecular conformations generated from the Zinc database. Positive molecules were batch-docked with the TRPV4 receptor protein (4DX2), and binding energy scores were calculated for each conformation. Screening results were ranked based on binding energy, and drug accessibility and potential antiviral effects were further analyzed. From the top 100 ranked molecules, 15 small-molecule candidates were selected for subsequent biological validation (Table S2). These candidates demonstrated potential as TRPV4 inhibitors, highlighting the combined efficacy of machine learning and molecular docking in identifying promising antiviral compounds (Table S2).

### 2.2. The Inhibitory Effect of the Drug Candidate on the Virus In Vitro

**Figure 3:**
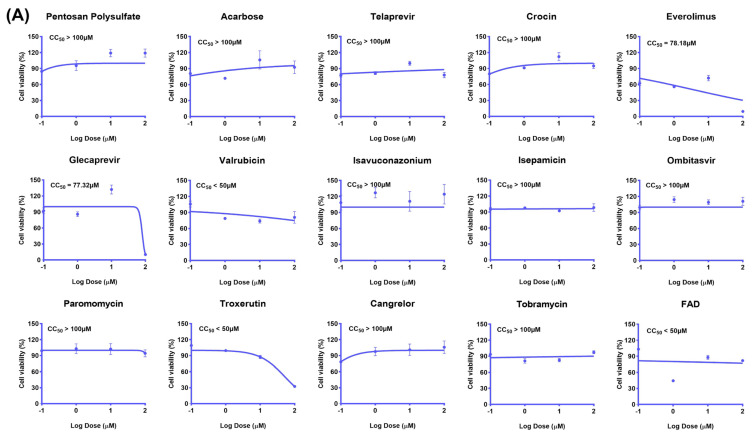

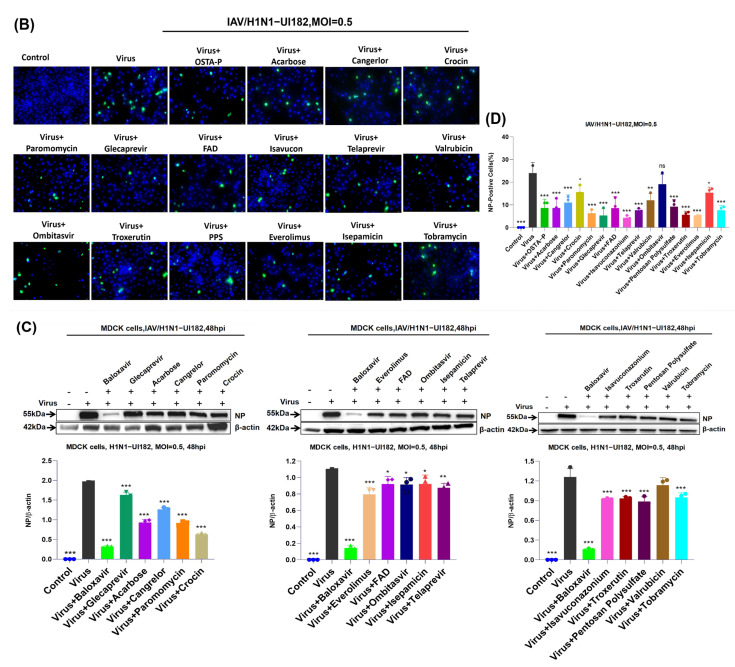
Viral inhibition of drug candidates in vitro. (**A**) In this figure, CCK−8 assay was used to detect the cytotoxic effect of selected drugs on MDCK cells. By staining drug-treated MDCK cells in response to CCK−8 reagents, the effects of the drugs on cell viability can be observed and quantified, and thus their potential cytotoxicity can be assessed. (**B**) In this figure, the expression of viral nucleoprotein (NP) in influenza virus-infected MDCK cells was examined using immunofluorescence after 48 h of drug incubation. By labeling the viral NP protein with a specific antibody and combining it with a fluorescently labeled secondary antibody, the distribution of the NP protein within the cell can be visualized under a microscope to understand the effect of the drug on viral replication. (**C**) Samples of the 15 selected drugs were collected 48 h after drug delivery and analyzed for H1N1-UI182 viral nucleoprotein (NP) expression and quantification using western blot images. (**D**) This figure shows the results of quantitative analysis of the immunofluorescence image obtained in (**B**). The results represented the mean of three independent experiments and were compared with a viral control group (±SD) (*n* = 3). *** *p* < 0.0001 for significant difference; ** *p* < 0.01 * *p* < 0.05 indicates.

In order to comprehensively confirm whether the screened drugs have potential antiviral effects, the toxicity inhibition effect of 15 drugs was tested first, and the screened drugs were diluted to four concentration gradients (0.1, 1, 10, 100 μM) respectively. Obviously, glecaprevir and everolimus showed significantly reduced cell activity at a maximum concentration of 100 μM, with median maximum cytotoxic concentrations (CC_50_) of 77.32 μM and 78.18 μM, which means that the two drugs may have toxic effects on MDCK cells at higher concentrations. To consider that different concentrations of the other drugs have little effect on MDCK cells, the subsequent experiments need to test the antiviral activity at the safe concentration of the drug (10 μM) to ensure that the virus can be effectively inhibited without causing significant damage to host cells (Figure 3A). To further evaluate the effects of 15 selected drug candidates on the expression of influenza virus protein (NP), oseltamivir phosphate (OSTA-P) and baloxavir were adopted as positive controls while IF and WB analysis were performed (Figure 3B,D). In IF assays, a significant decrease in the drug group was observed in viral protein (NP) expression which indicates that these drugs are effective in reducing viral replication and spread within cells (Figure 3B). In addition, WB results revealed 15 drugs that can attenuate the expression level of viral nuclear protein in different amplitudes, which further confirmed the above findings (Figure 3C). In summary, all 15 of the drugs screened showed the ability to inhibit the expression of viral protein (NP) in IF detection and WB analysis. These findings lay the foundation for our next step in vivo testing.

### 2.3. Evaluation of Candidate Drugs on Mice Infected with H1N1 Lethal Virus

To verify the antiviral effects of the 15 selected drug candidates fully in vivo, the virulent strain of influenza virus (HIN1−UI182) was used to infect an animal model (BALB/c mice) and examine the therapeutic effect of small molecule compounds. The experiments were divided into two separate batches based on the arrival time of the drug. To ensure the consistency of experimental conditions, the mice were inoculated with the same multiple of the toxic dose in each experimental batch. As per the preset dose, the drug candidates were administered while the corresponding positive control groups were set up for each drug group. The positive control group was given antiviral doses known to be effective (OSTA−P (25 mg/kg), baloxavir (5 mg/kg)) to compare the efficacy of the candidate drugs.

**Figure 4:**
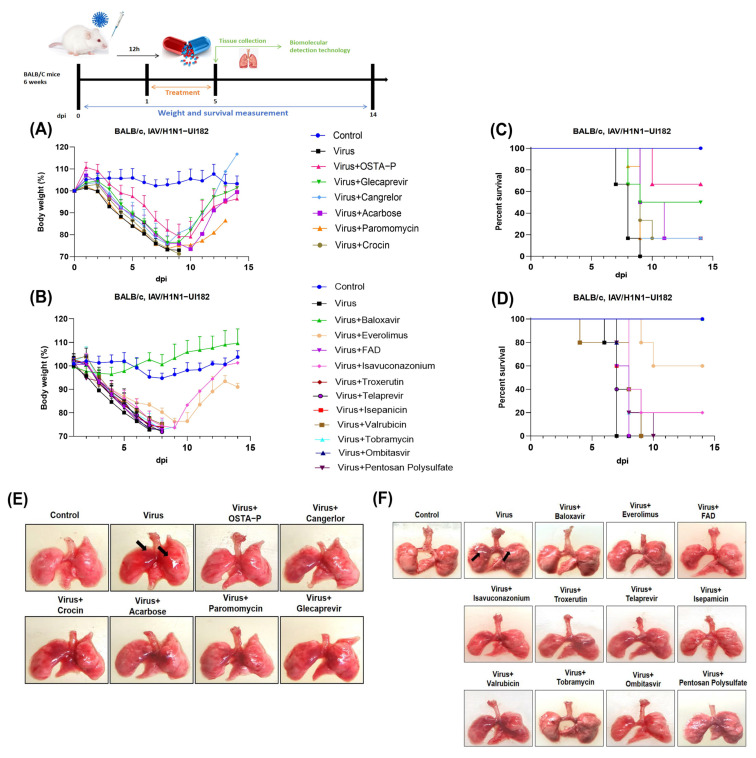

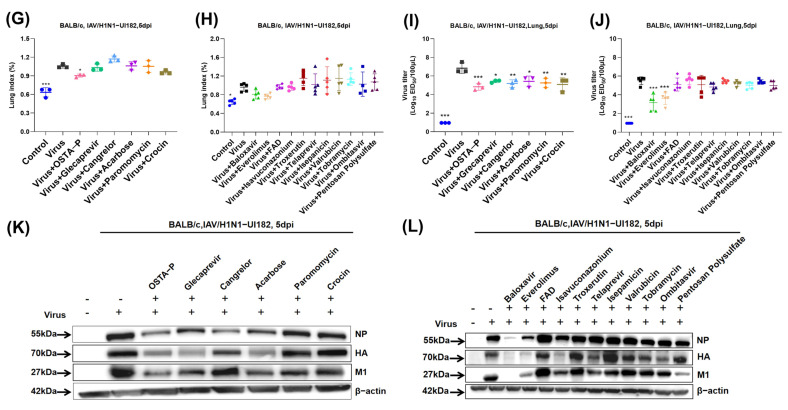
Drug candidate in mice infected with a lethal H1N1 virus. (**A**,**B**) Weight changes in mice infected with and treated with H1N1-UI182. (**C**,**D**) Survival status of mice infected with H1N1-UI182 and mice treated with drugs. (**E**,**F**) Lung pathology in mice infected with H1N1-UI182 and in mice treated with drugs 5 days after infection. (**G**,**H**) Changes in lung index of mice in the drug treatment group after 5 days of infection. The coloured lines with dots is error bar. (**I**,**J**) Changes of viral load in the lungs of mice in the drug treatment group 5 days after infection. (**K**,**L**) Western blot images showed the expression of viral nucleoprotein (NP), hemagglutinin (HA), and structural protein (M1). The results represented the mean of three independent experiments and were compared with a viral control group (±SD). *** *p* < 0.001 for significant difference; ** *p* < 0.01 * *p* < 0.05 indicates.

By monitoring the weight changes of the mice for 14 days, it was found that the mice in the virus infected group showed a noteworthy trend of weight loss from the first day after infection. Compared with the uninfected control group, the experimental group displayed a different degree of inhibition of viral replication. The combined results of the two studies showed that treatment with glecaprevir, cangrelor, acarbose, and everolimus significantly slowed the rate of weight loss in virus-infected mice (Figure 4A,B). Additionally, glecaprevir and everolimus significantly improved the survival rate of infected mice (Figure 4C,D). From the point of view of lung injury, all the mice in the administration group had obvious bleeding symptoms in the lungs from the fifth day after infection. Notably, mice in the glecaprevir and everolimus groups showed noticeable signs of lung improvement compared to other observed treatment groups (Figure 4E,F), especially the virus groups (Supplementary Figures S1A,B and S2A,B). Moreover, glecaprevir and everolimus significantly reduced viral titers, comparable to the positive drugs (Supplementary Figures S1C and S2C). The results of lung index and viral load implied that the viral load was evidently lower in the everolimus group compared to the viral group, suggesting that the drug was effective in reducing the degree of lung damage caused by viral infection (Figure 4G–J). Another positive finding is that expression levels of viral proteins in the treatment groups of glecaprevir and everolimus attenuated obviously referring to the results of WB bands. It indicates that the two drugs are able to effectively inhibit viral replication, thereby achieving the effect of reducing viral protein production (Figure 4K,L). In summary, these data emphasize that glecaprevir and everolimus not only effectively inhibited the replication of the virus in mice but also improved the survival rate and prolonged the survival time of mice infected with H1N1-UI182.

### 2.4. Protection of Glecaprevir and Everolimus at Different Concentrations in Mice Infected with H1N1 Lethal Virus

**Figure 5:**
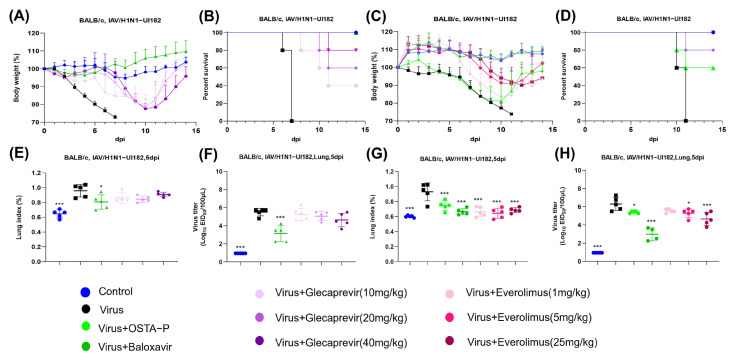

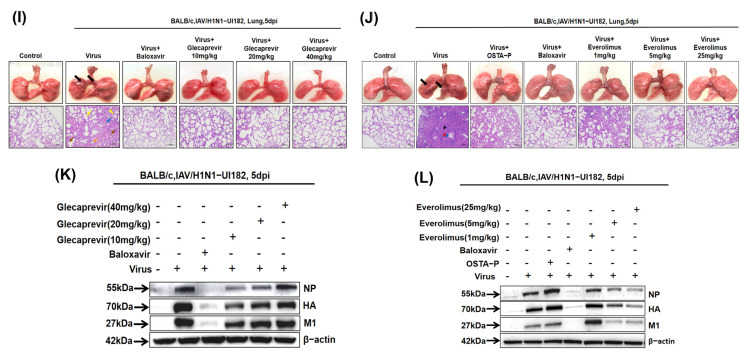
Glecaprevir and everolimus protect mice infected with H1N1 lethal virus. (**A**,**B**) Weight and survival trends in H1N1-UI182-infected mice and glecaprevir treated mice. (**C**,**D**) Weight and survival trends in H1N1-UI182-infected mice and everolimus-treated mice. (**E**) Changes of lung index in mice infected with H1N1-UI182 and treated with glecaprevir. (**F**) The effect of glecaprevir at 5 dpi on virus titer in the lung of mice was detected. (**G**) Changes of lung index in mice infected with H1N1-UI182 and treated with everolimus. (**H**) The effect of everolimus on virus titers in the lungs of mice at 5 dpi was detected. (**I**,**J**) Five mice in glecaprevir and everolimus groups were randomly selected 5 days after infection, and their lungs were dissected after euthanasia for examination by H&E staining analysis (*n* = 5). The yellow arrow represents granulocytes, the gray arrow represents the alveolar wall, the purple arrow represents alveolar dilation, the red arrow represents bleeding around the alveoli and blood vessels, the brown arrow represents epithelial cells, and the blue arrow represents macrophages. (**K**,**L**) Western blot images showed in glecaprevir and everolimus groups the expression of viral nucleoprotein (NP), hemagglutinin (HA) and structural protein (M1) in mouse lungs. The results represented the mean of three independent experiments and were compared with a viral control group (±SD). *** *p* < 0.001 for significant difference; * *p* < 0.05 indicates.

Combined with the previous results in vitro and in vivo, the screening criteria were determined as follows: (1) Pathological injury of the lungs after drug use; (2) The weight and survival of mice were improved markedly after the use of the drug. Thus, glecaprevir and everolimus are recognized for their effectiveness in suppressing the flu virus. To further assess the specific therapeutic effects of the two drugs on the lungs of mice infected with influenza virus at different concentrations, the experiment in vivo is necessary to determine whether there is a concentration dependence of the drugs. In this study, both drugs were administered orally to mice in the glecaprevir group (10 mg/kg/day, 20 mg/kg/day, and 40 mg/kg/day) and everolimus group (1 mg/kg/day, 5 mg/kg/day and 25 mg/kg/day); OSTA-P and baloxavir were selected as positive control drugs. The results demonstrated that infected mice treated with different concentrations of glecaprevir and everolimus observably retarded the rate of weight loss (Figure 5A–C) and improved survival in a concentration-dependent manner (Figure 5B–D) compared to mice treated with viral infection alone. This finding suggests that both drugs at different concentrations substantially improve weight loss and decrease survival due to viral infection.

In addition, according to the results of the lung index and pulmonary viral load of the glecaprevir group (Figure 5E,F), the inhibitory effect of glecaprevir on the virus did not display concentration-dependent characteristics. It can also be seen from the lung injury graph that higher concentrations did not alter the lung lesions in the mice (Figure 5I). It meant that the antiviral effect of glecaprevir at different concentrations was not significantly different. Correspondingly, WB results further confirmed that the drug did not effectively inhibit the expression of viral protein at higher doses but may promote the production of viral protein (Figure 5K). In contrast, the pulmonary index and pathological injury of the everolimus group (Figure 5G–J) indicated that the inhibitory effect of the drug on the virus was meaningfully dose-dependent, which means the inhibitory effect of the virus was enhanced with the increase of the dose of everolimus and that the pathological damage of the lung was reduced. To verify this finding, the pulmonary viral load of everolimus was measured at different concentrations (Figure 5H), and the results revealed that the pulmonary viral load decreased notably with the increase of drug concentration, which was equally proved by WB experiment (Figure 5L). It can be concluded that that appropriate adjustment of the dosage of everolimus can effectively control the degree of viral infection and in turn reduce lung inflammation and other related symptoms.

### 2.5. Molecular Mechanisms of Drug Candidates

**Figure 6:**
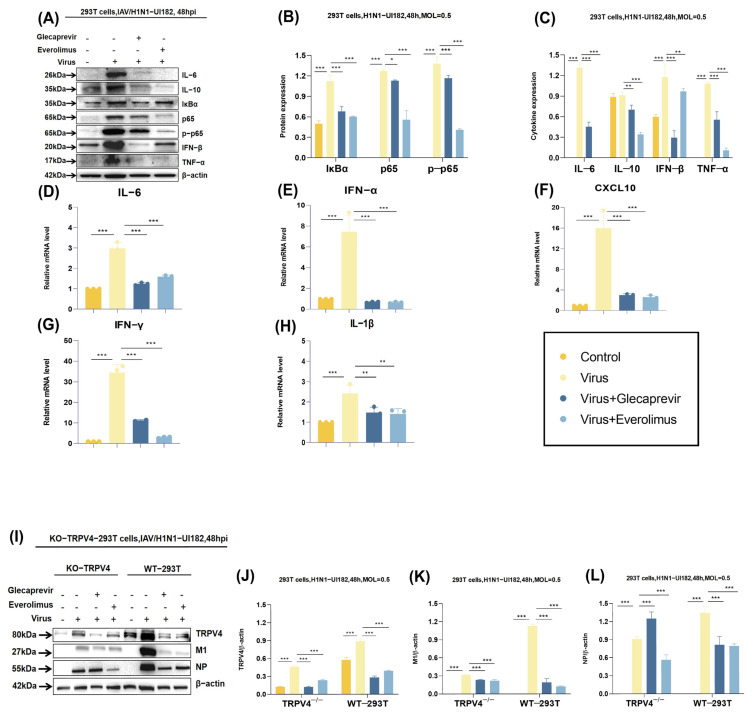
Glecaprevir and everolimus improved the inflammatory response as well as the expression of viral proteins after viral infection. (**A**–**C**) Western blot was used to detect the expression of cytokines and protein in the two drug groups. (**D**–**H**) Real-time fluorescence quantitative PCR (qPCR) was applied to detect the expression of cytokines in the two drug groups. (**I**–**L**) Western blot analysis of TRPV4, NP, and M1 expression after virus infection. The results represented the mean of three independent experiments and were compared with a viral control group (±SD) (*n* = 3). *** *p* < 0.001 for significant difference; ** *p* < 0.01 * *p* < 0.05 indicates.

To explain the roles of glecaprevir and everolimus in the inflammatory response induced by viral infection, we used WB and RT–qPCR to detect the expression levels of several key inflammatory cytokines. When influenza virus invades host cells, NF–κB can trigger a cascade of reactions that eventually lead to the release of NF–κB from its inhibitory protein IκBα into the nucleus, where it activates the transcription of multiple genes [19]. WB results showed a significant increase in the level of NF–κB phosphorylation in the case of infection with the H1N1–UI182 virus, suggesting that the virus activates the NF–κB signaling pathway (Figure 6A). Similarly, viral infection also induces the expression of several pro-inflammatory cytokines, including interleukin 6 (IL–6), interleukin 10 (IL–10), interferon β(IFN–β), and tumor necrosis factor α (TNF–α), all of which are significantly increased at the protein level (Figure 6A–C). However, under the treatment of glecaprevir and everolimus, the expression levels of all the above highly expressed cytokines decreased. Coincidentally, qPCR results showed that the mRNA expression level of infected virus group was specially up-regulated, while the mRNA expression level of chemokine CXCL10 also displayed a dramatic decrease in the cells treated with the two drug groups and mRNA expression levels of IFN–α, IFN–γ, IL–6, and IL–1β also showed a similar downward trend (Figure 6D–H). These results indicate that the two drugs may reduce the inflammatory response caused by infection with the H1N1–UI182 virus by inhibiting influenza virus replication and down-regulating the transcription levels of inflammatory cytokines. This finding provides important clues to understanding the underlying mechanisms of these two drugs in the treatment of H1N1 influenza virus infection.

To further determine the antiviral effect of glecaprevir and everolimus in TRPV4, both the 293T cells which were knocked out TRPV4 and normal 293T cells were adopted and added H1N1–UI182 virus with MOI = 0.5 to all cells except the blank control group. By detecting the expression of TRPV4, M1 and NP proteins, glecaprevir and everolimus were given to evaluate the effect of TRPV4. Results demonstrated that knocking out TRPV4 led to a significant decrease in the expression level of M1 protein, which inhibited the replication of H1N1 virus significantly (Figure 6I–K). Notably, the expression of M1 protein decreased more significantly in TRPV4^−/−^ 293T cells than in wild-type cells. This finding supports the notion that TRPV4 is critical in the life cycle of the H1N1 virus and may be a potential target for antiviral strategies. Interestingly, the expression level of NP protein was higher in normal cells after treatment with everolimus. On the contrary, the expression of NP protein was significantly decreased in TRPV4^−/−^ 293T cells (Figure 6L). This difference may be due to the fact that the inhibitory effect of everolimus on influenza virus is not only mediated through TRPV4, and there may be other targets involved. Collectively, the data confirm that glecaprevir and everolimus significantly improved the increased inflammatory response following viral infection as well as the expression of viral proteins, providing strong evidence for further exploration of the function of TRPV4 during the replication of the H1N1 virus and as a potential anti-influenza drug target.

### 2.6. Binding Mode of the Compounds with TRPV4 Receptor Protein

**Figure 7:**
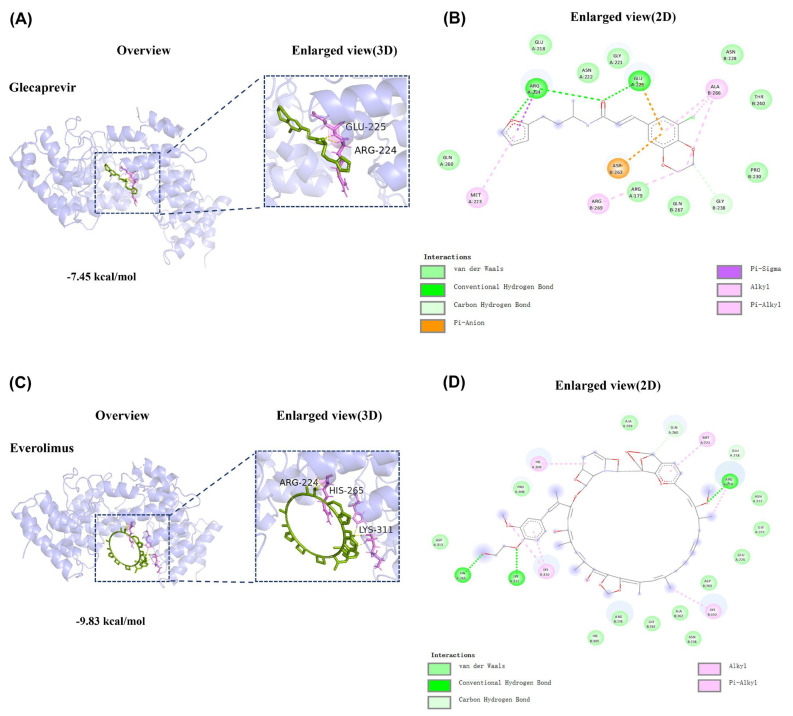
Binding mode of the compounds to 4DX2. (**A**,**B**) Binding mode of 4DX2 and glecaprevir. (**C**,**D**) Binding mode of 4DX2 and everolimus. Green cartoon represents the ligand compounds, purple sticks represent key residues in the covalent interaction, yellow dash lines represent hydrogen bonds. The corresponding 2D interaction maps are shown in (**B**,**D**) respectively, in which the hydrogen bond interactions are colored in dark green, with the van der Waals interactions in light green, the π−π stacking in dark pink, the π–alkyl interactions in light pink, and the π–anion and salt bridge interactions in orange.

A molecular docking analysis was conducted to investigate the interactions between 4DX2 and two drugs (glecaprevir and everolimus). Employing AutoDock version V1.5.7, both of the two docking results were generated and displayed low binding energies, indicating strong affinities between the compound and the target. Notably, glecaprevir forms a corresponding hydrogen bond with the 224 and 225 residues of the receptor protein, and everolimus interacts with receptor proteins at residues 224, 265, and 311 to form hydrogen bonds similarly. Both drug molecules show strong binding to the receptor protein with binding energies less than zero (glecaprevir: −7.45 kcal/mol, everolimus: −9.83 kcal/mol), which validates that the two drugs and receptor protein bind spontaneously, and they play an important role in the antiviral molecular mechanism through TRPV4 action. The visualizations of the lowest binding energies between target and compounds were created using Discovery Studio version 2.5 and Pymol. (Figure 7A–D)

## 3. Discussion

Recent studies have shown that TRPV4 channel is crucial in the process of influenza virus infection [20]. On the one hand, when influenza virus attempts to invade host cells, activation of the TRPV4 channel can promote an increase in the concentration of Ca^2+^ inside the cell. This increase in intracellular Ca^2+^ concentration facilitates the fusion of viral particles with the cell membrane, allowing entry into the cell interior [21]. Transient Ca^2+^ elevation is a key signal that may help regulate cell membrane fluidity and facilitate the fusion of virions with cell membranes which is crucial for the infection of the influenza virus [22]. On the other hand, the internalization of influenza virus usually involves the recombination of the cytoskeleton, especially the rearrangement of actin fibers [23]. The TRPV4 channel may also indirectly influence cytoskeletal dynamics by regulating intracellular Ca^2+^ levels, and this regulatory effect may contribute to the endocytosis of viral particles, which is the process by which viral particles are encased in the cell membrane to form vesicles and enter the cell interior. Once Ca^2+^ flows into cells, a series of downstream signaling events is also triggered [24]. These signaling events may further promote the transport of viral particles within the cell, providing the necessary environmental conditions for viral replication. Thus, the TRPV4 channel plays an essential role in facilitating early stages of influenza virus infection.

By inhibiting the activity of TRPV4 channels or knocking out TRPV4, it was hypothesized that the entry and replication process of the virus could be effectively prevented or slowed down, reducing the occurrence of infection. For example, compounds such as ruthenium red are able to effectively bind to the selective filter region of the TRPV4 channel, preventing it from opening and thus inhibiting the flow of Ca^2+^ into the interior of the cell [25]. Notably, it has been suggested that the administration of TRPV4 inhibitors can not only prevent the invasion of the virus but also improve the lung injury caused by the virus [26]. In brief, by inhibiting the TRPV4 channel, the infection rate of the virus can be effectively reduced, while the tissue damage and other complications caused by the virus can be ameliorated.

In this study, multiple machine learning models were also constructed to predict the activity of TRPV4 inhibitors, with the MACCS-SVM model demonstrating excellent performance and guiding the subsequent high-throughput screening. Subsequently re-screening was performed by referring to relevant literature in an attempt to lock in 15 compounds that perform antiviral activity in vitro. Moreover, we verified the effectiveness of two drugs, elecaprevir and everolimus, from 15 drugs in vivo, which not only effectively inhibited the replication of the virus, but also significantly improved the survival rate of infected mice (80% and 100% protection rates). It also regulates the inflammatory response and diminishes the production of inflammatory cytokines, thereby reducing the cell damage caused by viruses. It can be inferred that targeting TRPV4 to screen drugs is a new and effective way to treat drug-resistant strains of the virus.

The other molecular fingerprints (Morgan, RDKit (Greg Landrum, Switzerland), and AtomPairsFP) and classifiers (MLP, Random Forest, and XGBoost 2.0.0) also exhibited reasonable performance but did not surpass the MACCS-SVM combination in predictive accuracy. Notably, each fingerprint captures different molecular features, such as substructure patterns (MACCS), connectivity and circular patterns (Morgan), and atom pair relationships (AtomPairsFP), contributing unique perspectives on molecular activity. The principles underlying these differences suggest that certain fingerprints may align better with specific targets or datasets. For TRPV4 inhibitors, the success of MACCS-SVM may reflect the importance of specific substructural motifs that are effectively captured by MACCS keys and exploited by SVM’s hyperplane-based classification. While the MACCS-SVM model proved optimal in this study, future studies targeting other proteins or pathways should not disregard alternative combinations. Exploring a range of molecular fingerprints and classifiers remains essential to uncover novel insights and ensure robustness across different target systems.

Glecaprevir has been clinically proven to be a novel HCV NS3/4A protease inhibitor, which specifically inhibits the HCV NS3/4A protease to prevent the viral replication cycle [27]. Recent studies have found that it also performs antiviral activity against SARS-CoV-2 in addition to HCV [28], suggesting that it may be used as a potential treatment for SARS-CoV-2 infection. Although glecaprevir has rarely been reported to be used against influenza viruses, it has been proved to be safe and effective in the treatment of HCV [29]. In some studies, better antiviral effects can be achieved in human therapy if the original drug therapy concentration is increased for treatment. Unfortunately, the increase may also be accompanied by digestive discomfort (such as nausea, vomiting, diarrhea), allergic reactions, dizziness, and effects on liver or kidney function [30]. In order to minimize potential side effects, glecaprevir should be used in accordance with the recommended drug concentration and avoided self-adjustment of the drug concentration [31]. It is consistent with the opinion we promote that glecaprevir is not concentration-dependent in the treatment of mice infected with H1N1 lethal virus.

Everolimus is a potent, selective, and orally active mTOR1 inhibitor that clears damaged cells by inducing apoptosis and autophagy. Owing to its immunosuppressive properties, it is widely employed in transplantation medical research and cancer treatment [32]. Additionally, everolimus interferes with the replication of rumen-soluble bovine pox virus GLV-1 h68 [33] and is used in the treatment of HCV [34]. In this study, we found that glecaprevir and everolimus were not only effective against the above-mentioned viruses but also showed essential effects against influenza viruses (Figure 5A–L).

Although these findings suggest that glecaprevir and everolimus may have broader antiviral potential. However, this study also has certain limitations—that is, the specific mechanism of action of the drugs and their association with the TRPV4 target still require more in-depth research. Future studies need to comprehensively consider these factors in order to better exert the antiviral effects of these drugs and provide more powerful support for clinical treatment.

## 4. Materials and Methods

### 4.1. Machine Learning Models

In this study, we developed machine learning models to predict the activity of TRPV4 inhibitors based on molecular fingerprints. Molecular data for 506 TRPV4 inhibitors were collected from CHEMBL and PUBCHEM, represented as SMILES (simplified molecular input line entry system) (David Weininger, US) strings. To balance the data, 512 inactive molecules were randomly selected from PubChem, ensuring no overlap with known inhibitors and maintaining structural diversity through validation of molecular descriptors and fingerprints. These SMILES (David Weininger, US) strings were converted into various molecular fingerprints to capture structural and chemical features. The dataset was split into training and test sets in an 8/2 ratio using the train_test_split function from Scikit-Learn, ensuring representative distribution. This approach minimized bias and enhanced the model’s ability to distinguish between active and inactive molecules.

Five types of molecular fingerprints were generated: Molecular ACCess System (MACCS) keys; Morgan fingerprints (circular fingerprints based on the Extended Connectivity Fingerprints algorithm); RDKit fingerprints (chemical informatics library-based molecular descriptors). Multi-layer perceptron (MLP), support vector machine (SVM), random forest (RF) and extreme gradient boosting (XGBoost 2.0.0) were employed for model development. The best-performing model was selected based on prediction accuracy, and its scores were used for the large-scale virtual screening of candidate molecules, enabling the prioritization of compounds for docking and biological validation. To improve predictive performance, we selected four different machine learning models and optimized the hyperparameters for each model. First, the random forest (RF) model, an ensemble recursive partitioning method, was optimized using five key hyperparameters: the number of trees (n_estimators: 50; 100; 200); the splitting criterion (criterion: “gini” and “entropy”), the maximum tree depth (max_depth: none; 5; 10; 15); the minimum number of samples per leaf (min_samples_leaf: 1–10); and the number of features considered at each split (max_features: “log2”, “auto”, “sqrt”). The splitting criteria; “gini” (Gini impurity) and “entropy” (information gain); were employed to evaluate splits within decision trees. Gini impurity measures the likelihood of a randomly chosen element being incorrectly classified, while entropy quantifies the uncertainty or information content of a split. By constructing multiple decision trees from random subsets of the training data; the random forest model enhances both prediction accuracy and robustness.

Second, the support vector machine (SVM) model determines the optimal hyperplane in the feature space by maximizing the margin between classes, thus distinguishing objects with different labels. We optimized two hyperparameters for the SVM: the kernel function coefficient (gamma: “scale”, “auto”) and the penalty parameter for error terms (C: 0.01, 0.1, 1, 10). The SVM model is particularly suited for high-dimensional spaces and can handle nonlinear classification problems by mapping data to higher-dimensional spaces to find the optimal separating hyperplane.

Third, the multi-layer perceptron (MLP) is a type of artificial neural network used for complex pattern recognition. We adjusted hyperparameters including hidden layer sizes (hidden_layer_sizes: (50,), (100,), (50, 50)), activation functions (activation: “tanh”, “relu”), solvers (solver: “sgd”, “adam”), initial learning rates (learning_rate_init: 0.001, 0.01), and maximum iterations (max_iter: 10,000). The MLP captures complex patterns and relationships through multiple hidden layers and nonlinear activation functions.

Finally, the extreme gradient boosting (XGBoost 2.0.0) model, an advanced ensemble learning method based on the gradient boosting framework, was optimized with hyperparameters including maximum tree depth (max_depth: 3, 5, 7, 10, 15). The XGBoost 2.0.0 model improves performance by incrementally adding new trees to correct errors made by previous trees.

To prepare the docking molecules, the “FDA” and “world not FDA” (drugs approved, but not by the FDA) subsets from the Zinc database were chosen, resulting in 3447 molecules being filtered with the “for sale” criterion. The “for sale” filtering refers to the selection of compounds that are commercially available for purchase, ensuring practical accessibility for downstream experimental validation. After molecule deduplication, high-throughput virtual screening was performed using the best model scores as a guide. Additionally, Discovery Studio was employed to generate approximately 170,000 molecular conformations for batch docking with the TRPV4 receptor protein (PDB ID: 4DX2). The 4DX2 structure of TRPV4 was chosen due to its high resolution, which provides detailed atomic-level insights, and its relevance to ligand-binding studies. Among the available TRPV4 structures, 4DX2 specifically represents a biologically relevant conformation of the receptor in complex with a known ligand, making it an ideal candidate for docking and virtual screening. Consequently, the docking results were employed to calculate the binding energy, in the meanwhile, the optimal conformations of each molecular docking were taken and sorted by the calculation results. Ultimately, sort the drug molecules in the database from high to low in conformity with the calculation results. Since the results of molecular docking cannot distinguish between agonists and inhibitors and false positives also exist in computer simulations. Subsequently, biological verification is required.

### 4.2. Molecular Docking for Compounds and Target

The molecular docking approach was utilized to analyze the intermolecular interactions between 4DX2 and candidates in the paper through predictions of binding modes and affinities. The crystal structures of protein were downloaded from the RCSB Protein Data Bank (PDB). To remove water molecules and original ligands from the target protein, Pymol version 4.6.0 was adapted. Subsequently, the protein was imported to AutoDock Tools 1.5.7 for hydrogenation, charge calculation, and non-polar hydrogen combination. After determining ‘Grid Box’ size and genetic algorithm, AutoDock Vina version 1.2.5 was run for molecular docking. Finally, Discovery Studio and Pymol 4.6.0 were used to visualize the results.

### 4.3. Cell Culture, Reagents, Virus

Fetal bovine serum (FBS) and 10,000 U/mL penicillin–streptomycin solution, and Dulbecco modified Eagle culture medium (DMEM) were purchased from Thermo Fisher Scientific Corporation (Shanghai, China). A cell counting kit -8 (Beyotime, C0039, CCK-8) and dimethyl sulfoxide (DMSO) were acquired from Sigma-Aldrich (Shanghai, China). The virus used in this study was a mouse adapted strain (H1N1-UI182) of influenza A H1N1 2009 virus (A /Vinig/01/2009 (H1N1)). The H1N1 virus was inoculated in the Madin–Darby canine kidney cell line (MDCK) and human embryonic kidney cell line (293T). The virus and cells were stored in the Institute of Changchun Veterinary Research, Chinese Academy of Agricultural Sciences (Changchun, China). MDCK and 293T cell lines were cultured in DMEM with 10% fetal bovine serum (FBS, Gibco Lot.No.10438026) supplemented with 100 IU/mL penicillin and 100 μg/mL streptomycin.

The 15 candidate drugs covered in this article (CAS No. 42553-65-1; 163706-06-7; 56180-94-0; 1365970-03-1; 1263-89-4; 1258226-87-7; 67814-76-0; 7085-55-4; 946075-13-4; 5644-62-0; 32986-56-4; 37300-21-3; 159351-69-6; 402957-28-2; 146-14-5) were purchased from MCE Biotechnology Co. Ltd. (Zhangjiang Biopharmaceutical Base, Shanghai, China) and TargetMol Scientific Inc. (Pilot Free Trade Zone, Shanghai, China). As directed by the manufacturer, the medication was dissolved in dimethyl sulfoxide (DMSO), diluted in DMEM with 2% FBS or phosphate butylamine saline or dissolved with DMSO before use, and diluted with 2% fetal bovine serum or butyl phosphate (PBS, pH 7.4) DMEM.

### 4.4. Cytotoxicity Test and In Vitro Antiviral Activity Determination

MDCK cells were inoculated in 96-well plates at 37 °C and 5% carbon dioxide for 12 h. The cell supernatant was discarded when the cell density reached 60–70%, and then different concentrations of drugs (0.1, 1, 10, 100 μM) were inoculated in the cell plates, moreover 3 repeat holes were set for each concentration gradient. After 48 h, cell viability was measured using cell counting kit-8 (Beyotime, C0039, CCK-8) depending on factory instructions. The absorbance at 450 nm for each pore was measured using an enzyme-linked immunosorbent plate reader, and half inhibitory concentration (CC_50_) value of cell viability were calculated by a nonlinear regression curve fitting analysis (Prism 8) of cell viability to the log10 conversion concentration value.

### 4.5. Immunofluorescence Staining (IF)

The cells were seeded in 12-well plates and infected with H1N1/UI182 (MOI=0.5) when confluence reached 70%. At 48 h post-infection, the medium in the cell pore was discarded and washing with phosphate buffered saline (PBS, P1020, Solabio, China) was repeated 2–3 times. Fixed with 4% paraformaldehyde (PFA) for 30 min, the membrane was permeated with 0.5% Triton X-100 (P0096, Beyotime, China) for 15 min. Subsequently, the cells were blocked with 2% bovine serum albumin (BSA, 9048-46-8, Sigma, St. Louis, MA, USA) and added to anti-influenza nucleocapsid protein primary antibody (Abcam, ab104870, 1:400). After incubation at 4 °C overnight, the cells were washed with PBS 3 times and incubated with secondary antibodies (goat anti-rabbit IgGH&L (AlexaFluor^®^ 488), Abcam, ab150077, 1:500) for 2 h under no-light conditions. Finally, staining with Hoechst 33,258 (Thermo Fisher Scientific fc, H3569,1 µg/mL) for 10 min was observed and recorded under a fluorescence microscope (APX100, Olympus, Shinjuku-ku, Tokyo, Japan).

### 4.6. Infection in Mice and Determination of Lung Index

Healthy mice aged 6–8 weeks (female, BALB/c, weight 15–20 g) selected by Beijing Vitonglihua were feed adapted for one week in a BSL-2 laboratory and divided into the untreated group (blank group), viral group, positive control group, and compound treatment group. A mouse adaptive strain of influenza virus diluted to 5 × LD_50_ was inoculated with a dose of 50 μL in the virus group and the drug treatment group via nasal drip, and the administration began 12 h after infection. The blank control group and the viral infection group were treated with 0.9% normal saline (i.p.) by oral gavage; the positive control group was treated subcutaneously with Baloxavir (5 mg/kg/d) or orally with oseltamivir phosphate (25 mg/kg); while the drug treatment group was treated every day (oral gavage/intrabitoneal injection, once in the morning and once in the evening). Then, the host body weight and survival rate before and after the compound treatment were detected and recorded. On the fifth day post-infection (5 dpi), lung tissues of mice were collected to detect lung index, viral titer in lung tissue, local histopathological changes, and viral protein expression in the lungs. Clinical signs related to weight change, survival, and disease were observed until 15 dpi.

### 4.7. RNA Isolation and Quantitative RT-qPCR

To extract total RNA from mouse lung tissue, the HiPure universal RNA extraction kit (R4130-03, Magen, China) was employed and subsequently the nano-photometer (Thermo Fisher Scientific, Shanghai, China) was used for measuring. In accordance with the manufacturer’s instructions, total RNA was reverse-transcribed into cDNA using a priscript™RT kit (Takara, RR047A, Otsu City, Shiga Prefecture, Japan). At last, the RT-qPCR system (Bio-Rad, C1000 Touch™ Thermal Cycler, Hercules California, CA, USA) was used to quantitatively express genes against β-actin. The sequence of primers is shown in Table S1.

### 4.8. EID_50_ Detection

To detect EID_50_, 10-day-old SPF-grade chicken embryos were adopted. After mouse lung tissues stored in the refrigerator at −80 °C were removed and sterile small steel balls and DMEM culture solution containing diamantine were added to the centrifuge tube and ground at 30 Hz for 6 min. Followed by centrifugation, the supernatant was serially diluted 10-fold from 10^−1^ to 10^−8^ (3 repeats per gradient), and pipette 100 μL into each group of 3 10-day-old SPF eggs, and sealed with paraffin wax; 48 h later, 1% washed chicken red blood cells (RBC) were used as the index system. Then 50 μL allantoic fluid in each egg was collected and mixed with 50 μL 1% chicken RBC. The samples were placed at room temperature for 15 min, whereafter the hemagglutination results of chicken embryos were measured. The viral titer was calculated using the Reed–Muench method, and the result was expressed as log10EID_50_.

### 4.9. Pathological Analysis

On the third and fifth days after infection, three mice from each group were euthanized to collect tissues, and the tissues were fixed with 4% paraformaldehyde (PFA) at room temperature for hematoxylin and eosin (H&E) staining. The fixed tissues were dehydrated in ethanol and embedded in paraffin. The processed tissues were sectioned and stained H&E with solution (Thermo Fisher Scientific, Debao Road, Shanghai, China), and the sections were scanned using a digital microscope (Olympus, Shinjuku-ku, Tokyo, Japan). The pathological score was summarized based on hemorrhage, thickening of alveolar walls, inflammation, and cell necrosis.

### 4.10. Western Blot (WB)

Cell samples and mouse tissues were lysed in RIPA lysis buffer (P0013B, Beyotime, Tongnan Road, Shanghai, China) containing proteinase inhibitor, and the protein concentration was determined using a BCA protein detection kit (P0010S, Beyotime, Tongnan Road, Shanghai, China). For each sample, 10–15 µg protein was loaded on an SDS polyacrylamide gel and fractionated by electrophoresis. Separated proteins were transferred to PVDF membranes (Thermo, China) and blocked with 5% BSA. Subsequently, the membrane was incubated with primary antibodies and horseradish peroxidase (HRP)-conjugated secondary antibodies. Finally, proteins were detected using the chemiluminescent image system (Tanon, Jiading District, Shanghai, China), while the ImageJ software (NIH, Bethesda, MD, USA) was used for grayscale analysis and quantification with β-actin as the internal reference protein.

The following antibodies were used: NP(Abcam, ab104870, 1:1000), β-actin (Abcam, ab6276, 1:500), HA(GeneTex, HL3261), M1(GeneTex, HL1275), IL-10(CST, D13A11), IL-6(CST, D5W4V), TNF-α(CST, D2D4), IκBα(CST, 44D4), IFN-β(CST, D2J1D), p65(CST, D14E12), p-p65(CST, 93H1), goat anti-rabbit IgG (Beyotime, A0208, 1:1000), and goat anti-mouse IgG (Beyotime, A0216, 1:1000).

### 4.11. Statistical Analysis

All statistical analyses were performed using GraphPad Prism 8.0.2 (GraphPad Software, Cambridge, UK). One-way ANOVA or Student’s *t* test was used to determine significant differences. Data are represented as means ± standard deviations. In all cases, a *p* value < 0.05 is considered to be significant, and * *p* < 0.05, ** *p* < 0.01, *** *p* < 0.001.

## 5. Conclusions

A high-throughput virtual screening with TRPV4 as the target, combined with biological experimental verification and machine learning model evaluation, identified glecaprevir and everolimus as effective inhibitors of influenza virus infection both in vitro and in vivo. These drugs significantly improved the survival rate of mice infected with H1N1-UI182 and mitigated inflammatory factors. Further analysis confirmed that the antiviral effect of these two drugs may affect the replication of the influenza virus by inhibiting the expression of TRPV4. The integration of machine learning, specifically the MACCS-SVM model, into the drug screening process significantly enhanced the efficiency and accuracy of identifying promising candidates. Taken together, this work highlights the utility of repurposing drugs as antivirals and provides new ideas for the research of anti-influenza virus drugs.

## Data Availability

Dataset available on request from the authors.

## Supplementary Materials

The following supporting information can be downloaded at: https://www.mdpi.com/article/10.3390/ijms26031381/s1.

## Abbreviations

293T	Human embryonic kidney 293T cells
AUC	Area under the curve
BA	Balanced accuracy
BSA	Bovine serum albumin
CADD	Computer-aided drug design
CC_50_	Maximum cytotoxic concentration
CCK-8	Cell counting kit-8
DPI	Days post-infection
DMSO	Dimethyl sulfoxide
DMEM	Dulbecco modified Eagle culture medium
EID_50_	50% egg infectious dose
FBS	Fetal bovine serum
H&E	Hematoxylin and eosin
HRP	Horseradish peroxidase
HSV-2	Herpes simplex virus type 2
IV	Influenza virus
IAV	Influenza A virus
IL-6	Interleukin 6
IL-10	Interleukin 10
IFN-β	Interferon β
MDCK	Madin–Darby canine kidney
M1	Matrix protein 1
mTOR1	Mammalian target of rapamycin complex 1
MLP	Multi-layer perceptron
MCC	Matthews correlation coefficient
MACCS	Molecular access system
NP	Nucleoprotein
PA	Polymerase acidic
PDB	Protein data bank
PFA	Paraformaldehyde
RBC	Red blood cells
RT-qPCR	Real-time quantitative fluorescent PCR
RF	Random forest
SPF-grade	Specific pathogen free-grade
SMILES	Simplified molecular input line entry system
SVM	Support vector machine
TRPV4	Transient receptor potential vanilloid 4
TNF-α	Tumor necrosis factor α
TPSA	Topological polar surface areas
WB	Western blot
XGBoost	Extreme gradient boosting
