# Supplementary material for: Discovery of TRPV4-Targeting Small Molecules with Anti-Influenza Effects Through Machine Learning and Experimental Validation
Source: Int J Mol Sci. 2025 Feb 6;26(3):1381. doi: 10.3390/ijms26031381 (PMC11818416; doi:10.3390/ijms26031381)
Supplement: Supplementary file 1 [file ijms-26-01381-s001.zip › ijms-3408675-supplementary.pdf]

Supplementary material

Appendix S

Table S1. Primer sequences for PCR.

Gene name	Primer sequence(5 ’to 3’)
β-actin(mouse)	F :5'-TGACGTTGACATCCGTAAAGACC-3’
	R : 5'-AAGGGTGTAAAACGCAGCTCA-3’
IFN-a(mouse)	F : 5'-GCACCCTGCCTCAGACTCAC-3’
	R : 5'-TGCCTGGTCATCTCATGGAAG-3’
IL-1B (mouse)	F : 5'-TCATCGTGGCAGTGGAAAAG-3’
	R : 5'-GGGAAGCAAGGGTCTCAGGT-3’
IL-6 (mouse)	F : 5'-AGTTGCCTTCTTGGGACTGATG-3’
	R : 5'-GGGAGTGGTATCCTCTGTGAAGTCT-3’
IFN- γ (mouse)	F : 5'-AGCCAAATCGTCTCCTTCTACTTC-3’
	R:5'-TGCACCTTGTTGCTGCTGTT-3’
CXCL-10(mouse)	F : 5'-ATGAGCCTGACCTGCTGTCT-3’
	P : 5'-TTACTCCACGCTTCCTGCTG-3’

Table S2. Computer virtual screening results for the top 100 compounds.

No.	ZINC-ID	Binding energy	Name	CAS_ID	Avail-ability	Antivi-ral ef-fect
1	ZINC000245224178	-472.465	CROCIN	42553-65-1	yes	yes
2	ZINC000252286875	-360.011	Nystatin	1400-61-9	no	no
3	ZINC000008215434	-329.664	Fad	146-14-5	yes	yes
4	ZINC000245190613	-322.817	Nystatin	1400-61-9	yes	no
5	ZINC000252286877	-319.366	Amphotericin A	1405-32-9	no	no
6	ZINC000085537017	-319.091	Cangrelor	163706-06-7	yes	yes
7	ZINC000169289419	-311.858	Carbetocin	37025-55-1	yes	no
8	ZINC000096309558	-309.229	Acarbose	56180-94-0	yes	yes
9	ZINC000252286878	-306.791	Nystatin	1400-61-9	no	no
10	ZINC000150338698	-306.644	Ethyl ketone	65236-63-7	no	no
11	ZINC000008551087	-304.5	Coenzyme	85-61-0	yes	no
12	ZINC000169289386	-296.881	Saralasin	39698-78-7	yes	no
13	ZINC000252441679	-294.431	Nystatin	1400-61-9	no	no
14	ZINC000085537042	-293.425	Acarbose	56180-94-0	yes	no
15	ZINC000245190611	-286.158	Nystatin	1400-61-9	no	no
16	ZINC000936069565	-285.699	Glecaprevir	1365970-03-1	yes	yes
17	ZINC000008214644	-282.027	Pentagastrin	5534-95-2	yes	no
18	ZINC000013556853	-281.895	Lamivudine-Tri-phosphate	147217-71-8	no	no
19	ZINC000095618609	-278.828			no	no
20	ZINC000006920404	-278.547	Maltotriose	1109-28-0	yes	no
21	ZINC000060183170	-278.228	Paromomycin sulfate	1263-89-4	yes	yes
22	ZINC000085551979	-276.872	Nystose	13133-07-8	no	no

23	ZINC000253387843	-276.312	Amphotericin B	1397-89-3	no	no
24	ZINC000238850852	-276.253	Eptifibatide	188627-80-7	no	no
25	ZINC000033753205	-275.916	6-T-GDP	16541-19-8	no	no
26	ZINC000087496092	-274.804	Acarbose	56180-94-0	no	no
27	ZINC000008552309	-272.523	1-Palmitoyl-2-oleoyl-sn-glyc-ero-3-phosphati-dylglycerol	185435-28-3	no	no
28	ZINC000242548690	-270.534	Digoxin	20830-75-5	yes	no
29	ZINC000252286876	-270.295	Nystatin	1400-61-9	no	no
30	ZINC000253668332	-269.753	Deslanoside	17598-65-1	yes	no
31	ZINC000256097222	-268.993			no	no
32	ZINC000030731261	-268.942	6-Thioinosine Triphosphate	27652-34-2	yes	no
33	ZINC000150601177	-267.527	Ombitasvir	1258226-87-7	yes	yes
34	ZINC000004261765	-267.456	Atp	34369-07-8	yes	no
35	ZINC000016051982	-267.183	Gemcitabine Di-phosphate	35846-53-8	yes	no
36	ZINC000022446966	-262.636	Gadavist	770691-21-9	yes	no
37	ZINC000261106252	-262.617	C261106252	106128-89-6	no	no
38	ZINC000008214585	-261.659	Isepamicin	58152-03-7	yes	yes
39	ZINC000085552699	-261.21	Thr	7085-55-4	yes	no
40	ZINC000049799668	-260.55	Viomycin	32988-50-4	no	no
41	ZINC000096014306	-258.213			no	no
42	ZINC000096006021	-258.045	Josamycin	16846-24-5	no	no
43	ZINC000003870129	-257.626	Oxiglutatione	103239-24-3	yes	no
44	ZINC000072206342	-256.454	Rutin	153-18-4	yes	no
45	ZINC000049637509	-255.13	Isavucona-zonium	946075-13-4	yes	yes
46	ZINC000011616852	-253.315	Valrubicin	56124-62-0	yes	yes
47	ZINC000004217203	-251.931	Oftasceine	1461-15-0	yes	no
48	ZINC000003917540	-250.726	Dalfopristin	112362-50-2	yes	no
49	ZINC000085537068	-250.358			no	no
50	ZINC000028232755	-250.27	Valrubicin	56124-62-0	yes	yes
51	ZINC000169362009	-250.12	Atosiban	90779-69-4	yes	no
52	ZINC000013546270	-249.836	Gemcitabine Tri-phosphate	110988-86-8	yes	no
53	ZINC000008214383	-249.329	Dibekacin	58580-55-5	yes	no
54	ZINC000008214692	-244.919	Tobramycin	32986-56-4	yes	yes
55	ZINC000043131420	-244.595	Fostamatinib	901119-35-5	yes	no
56	ZINC000003830957	-244.375	Iopromide	73334-07-3	yes	yes
57	ZINC000094437910	-244.157	1,2-dimyristoyl-Sn-glycerol-3-phosphate - SN-glycerol sodium salt	116870-30-5	yes	no
58	ZINC000014879975	-243.119	Pentosan Poly-sulfate	37300-21-3	yes	yes
59	ZINC000003830946	-242.46	Iohexol	66108-95-0	yes	no
60	ZINC000002005305	-242.067	Methyltetrahy-drofolic acid	134-35-0	yes	no
61	ZINC000054053579	-241.994	Dalfopristin	112362-50-2	yes	no

62	ZINC000068150640	-241.213	Plazomicin	1154757-24-0	yes	no
63	ZINC000008215403	-239.826	Dpnh	606-68-8	yes	no
64	ZINC000071928291	-238.437	Neomycin	1405-10-3	yes	no
65	ZINC000061389419	-238.076	Ribostamycin	25546-65-0	yes	no
66	ZINC000256097213	-237.257	Hydroxyethyl cellulose	9004-62-0	yes	no
67	ZINC000150338703	-236.28	Carbetocin	37025-55-1	yes	no
68	ZINC000169289411	-235.898	Tacrolimus	104987-11-3	yes	yes
69	ZINC000060392785	-235.701			no	no
70	ZINC000254113657	-235.117	Everolimus	159351-69-6	yes	yes
71	ZINC000256097218	-234.79	Hydroxyethyl cellulose	9004-62-0	yes	no
72	ZINC000885764928	-234.509	Paritaprevir	1216941-48-8	yes	no
73	ZINC000263621850	-233.551	4-Glutathionyl Cyclophospha-mide	50-18-0	no	no
74	ZINC000242437513	-233.475	Gentamicin	26098-04-4	yes	no
75	ZINC000238809655	-233.27	Rg1 Ginsenoside Rg1	22427-39-0	yes	no
76	ZINC000003860156	-231.782	Amp	18422-05-4	yes	no
77	ZINC000060183177	-230.372	Fungichromin)	6834-98-6	yes	no
78	ZINC000242437512	-230.222	Gentamicin	26098-04-4	yes	no
79	ZINC000085537142	-230.109	Aclarubicin	57576-44-0	yes	no
80	ZINC000003992480	-229.994	Telaprevir	402957-28-2	yes	yes
81	ZINC000253855706	-229.215	Josamycin	16846-24-5	yes	no
82	ZINC000028232746	-227.862	Valrubicin	56124-62-0	yes	yes
83	ZINC000096015174	-227.678	Glycyrrhizinate Dipotassium	1405-86-3	yes	no
84	ZINC000085552114	-227.601	MALTO-TETRAOSE	34612-38-9	yes	no
85	ZINC000004228266	-227.519	Methyl Folate	134-35-0	yes	no
86	ZINC000203686879	-227.4	Velpatasvir	1377049-84-7	yes	no
87	ZINC000009575047	-226.831	Arbekacin	51025-85-5	yes	no
88	ZINC000003830944	-225.895	IOHEXOL	66108-95-0	yes	no
89	ZINC000150338506	-225.52	Inositol Niacin-ate	6556-11-2	yes	no
90	ZINC000003938704	-224.54	Epirubicin	56420-45-2	yes	no
91	ZINC000253633751	-224.457	Natamycin	7681-93-8	yes	no
92	ZINC000242437514	-223.549	Gentamicin	26098-04-4	yes	no
93	ZINC000003830958	-222.861	Iopromide	73334-07-3	yes	no
94	ZINC000003875881	-222.753	Fluorodeoxyuri-dylate	71963-69-4	yes	no
95	ZINC000009212425	-222.274	Folinic acid	58-05-9	yes	no
96	ZINC000095862733	-221.835	Digitoxin	71-63-6	yes	no
97	ZINC000019855121	-219.942	Ethylenebis(ni-trilodi-methylene)tetra-phosphonic acid	13528-93-3	no	no
98	ZINC000029571072	-219.763	Isavucona-zonium	946075-13-4	yes	yes
99	ZINC000024782119	-219.758	Dimyristoyl phosphatidyl-choline	18656-38-7	yes	no

100	ZINC000003831231	-219.615	Novobiocin sodium	1476-53-5	yes	yes

Two additional conditions are taken into account: 1) availability; 2) antiviral effect. According to these two criteria, 15 compounds requiring further verification were selected.

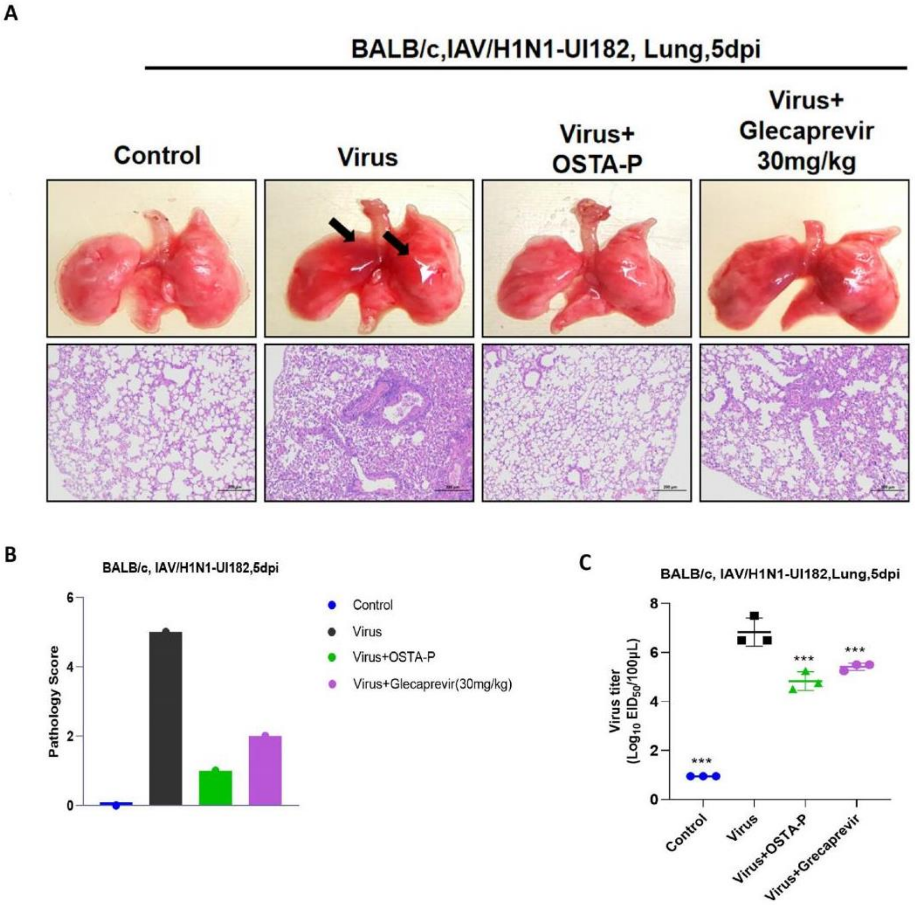

Figure S1. Glecaprevir protects the lungs. **(A)** The process of lung tissue dissection in mice treated with 30 mg/kg glecaprevir was conducted on the fifth day of the experiment. Images of the staining results were obtained by processing these lung tissue samples with the classic H&E (hematoxylin - eosin) staining technique. These images clearly show the effect of glecaprevir on the lung tissue of mice at this dose. The black arrows represent the pulmonary lesions in the virus group. **(B)** Pathological scoring of glecaprevir - treated lung sections was carried out after processing. **(C)** Differences in lung viral load were analysed using glecaprevir versus control.

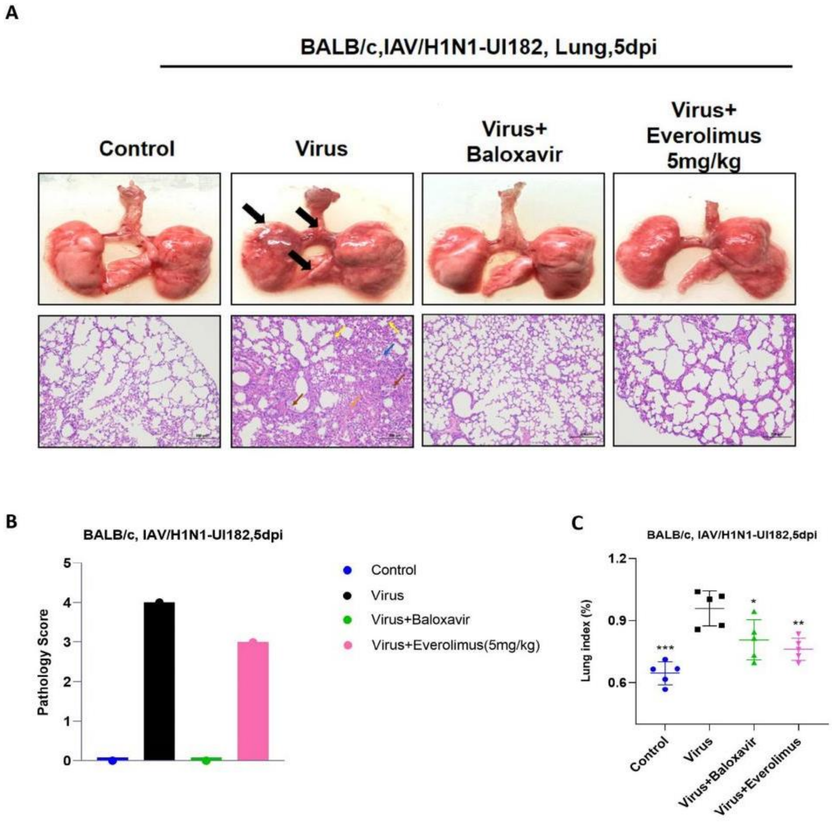

Figure S2. Everolimus protects the lungs. **(A)** The process of lung tissue dissection in mice treated with 5 mg/kg everolimus was carried out on the fifth day of the experiment. Images of the staining results were obtained by processing these lung tissue samples with the classic H&E (hematoxylin - eosin) staining technique. These images clearly show the effect of everolimus on the lung tissue of mice at this dose. **(B)** Pathological scoring of everolimus - treated lung sections was carried out after processing. Granulocytes (yellow arrows), alveolar walls (grey arrows), alveolar dilation (purple arrows), perivascular hemorrhage (red arrows); epithelial cells (brown arrows); macrophages (blue arrows). **(C)** The effect of everolimus on lung indices was analysed by comparing it with the control group.
